# Phytochemical Screening and Antimicrobial Activity Evaluation of Armenian *Chaerophyllum bulbosum* L.

**DOI:** 10.3390/molecules31162806

**Published:** 2026-08-12

**Authors:** Neli Ghukasyan, Naira Shaboyan, Elena Arutinian, Arshaluys Ghazaryan, Vahe Hovhannisyan, Gayane Poghosyan, Marine Hovhannisyan, Kostandin Manukyan, Greta Ulikhanyan, Sona Feschyan, Maya Hovsepyan, Lusine Danielyan, Hrachya Hovhannisyan, Naira Chichoyan, Serkos Haroutounian

**Affiliations:** 1 Department of Pharmacognosy, Yerevan State Medical University, 2 Koryun St., Yerevan 0025, Armenia; nellka2011@gmail.com (N.G.); arshaluysghazaryan11@gmail.com (A.G.); vahehovhannisyan_88@mail.ru (V.H.); 2 Zueripharm AG, Suedstrasse 3, 8952 Schlieren, Switzerland; n.shaboyan7@gmail.com; 3 Faculty of Stomatology, Yerevan State Medical University, 2 Koryun St., Yerevan 0025, Armenia; eharutinyan@gmail.com; 4 Department of Medical Microbiology, Yerevan State Medical University, 2 Koryun St., Yerevan 0025, Armenia; gayanep5@yandex.ru (G.P.); marinahov5@yandex.ru (M.H.); kostandinmanukyan@yandex.ru (K.M.); 5 Department of Medical Physics, Yerevan State Medical University, 2 Koryun St., Yerevan 0025, Armenia; gretaulikhanyan@gmail.com; 6 Department of Medical Biology, Yerevan State Medical University, 2 Koryun St., Yerevan 0025, Armenia; feschyan.sona@mail.ru (S.F.); maia.hovsepyan@gmail.com (M.H.); 7 Scientific & Production Center “Armbiotechnology”, NAS of Armenia, 14, Gyurjyan Str., Yerevan 0056, Armenia; 20mar02@mail.ru (L.D.); hhov@sci.am (H.H.); 8 Laboratory of Nutritional Physiology & Feeding, Department of Animal Science, Agricultural University of Athens, Iera Odos 75, 11855 Athens, Greece; sehar@aua.gr

**Keywords:** *Chaerophyllum bulbosum* L., essential oil, hydrosol, pulegone, antimicrobial activity

## Abstract

This study is the first report on the chemical composition and antimicrobial activity of *Chaerophyllum bulbosum* L. sampled from the Armenian flora. Essential oil (EO) and hydrosol were obtained from the aerial parts by hydrodistillation, and their chemical compositions were determined by GC–MS, identifying a total of 48 volatiles. The EO was composed of 60.42% monoterpenes and their oxygenated derivatives and 18.75% sesquiterpenes and their oxygenated derivatives, while the hydrosol was composed of 75% oxygenated monoterpenes, with the remaining 20% and 5% being monoterpenes and sesquiterpenoids respectively. Pulegone (21.55%) was identified as the major constituent. With regard to the non-volatile fraction, the methanolic extract displayed a total phenolic content of 1.17 ± 0.03 mg GAE/g dry weight and a total flavonoid content of 0.62 ± 0.02 mg QE/g, as well as significant antioxidant capacity, with IC_50_ values of 2.87 ± 0.06 μg Trolox/g dried plant sample for the DPPH^•^ assay and 46.87 ± 1.2 μmol Fe^2+^/g dried plant sample for the FRAP assay. The evaluation of EO and hydrosol antimicrobial properties revealed notable antibacterial activity against Gram-positive bacteria, with inhibition zones ranging from 13.5 to 20 mm. The respective minimum inhibitory concentration (MIC) values ranged from 0.4 to 6.0 mg/mL for the EO and from 62.5 to 400 μL/mL for hydrosol, while the minimum bactericidal concentration (MBC) values ranged from 0.8 to 10.0 mg/mL and from 125 to 800 μL/mL, respectively. *Streptococcus mutans* was the most susceptible microorganism (MIC = 0.4 mg/mL), whereas *Pseudomonas aeruginosa* was the most resistant (MIC = 6.0 mg/mL). Both the EO and hydrosol exhibited antifungal activity against *Candida albicans*, with inhibition zones of 13.0 ± 1.2 mm and 11.0 ± 0.8 mm, respectively. The MIC and MBC values were 1.0 and 2.0 mg/mL for the EO, while the respective values for the hydrosol were 400 and 800 μL/mL. These findings highlight the *C. bulbosum* plant as a promising source of bioactive compounds with significant activity against Gram-positive bacteria and moderate antifungal effects that may be associated with the high content of pulegone, a monoterpene known for its membrane-disruptive properties.

## 1. Introduction

Plant-based foodstuffs constitute an important source of various vitamins, minerals, fibers, fatty acids and health-promoting bioactive molecules such as phenolic acids, flavonoids and terpenoids [1]. In this respect, numerous edible plants have served as valuable natural resources, displaying a rich history of traditional use. Their therapeutic properties, from fighting infections to treating chronic diseases, underscore their importance in modern medicine. The presence of key bioactive compounds further increases their potential in the pharmaceutical and food industries. Among the most well-studied plants of nutritional importance are those belonging to the Apiaceae (Umbelliferae) plant family, which constitutes one of the largest plant families with worldwide distribution, although most of them originate from countries in the Mediterranean basin. Within this family, approximately 450 genera and 3700 species are classified [2,3,4], with many plants known as a good source of secondary metabolites exhibiting diverse biological activities. Some of their health benefits relate to their ability to induce apoptosis, as well as antibacterial, hepatoprotective, vasorelaxant, cyclooxygenase-inhibitory and antitumor activities. Additionally, several studies reveal the potent biocidal activities of Apiaceae plant species, expressed as insecticidal, herbicidal and antifungal activities [5,6].

Previous scientific publications often concern the study of common, endemic species of Umbelliferae plants in Northeast Anatolia, such as *C. karsianum* and *C. posofianum*, mainly focusing on the anatomical and micromorphological characteristics of their fruits or on their nucleotide sequence data, which show that the internal transcribed spacer sequences of *C. karsianum* and *C. posofianum* are identical to those of *C. bulbosum* [7]. According to a literature finding, *C. bulbosum* collected from Ardahan (Armenia) is a potent source of natural phytochemicals for the development of phytopharmaceuticals focusing on oxidative stress-related diseases. Moreover, it has been confirmed that the polar extracts possess significant antioxidant activity because of their high amounts of phenols and flavonoids [8,9].

Another scientific endeavor concerning a bulbous chervil species growing in Serbia compared the chemical composition and the antimicrobial and antioxidant activities of essential oils (EOs) isolated from the roots and fresh aerial parts of *C. bulbosum.* The results indicated that EO from aerial parts is composed of 90% (*E*)-*β*-ocimene, underlying its valorization potential by the perfumery industry. Concomitantly, it was shown that the antibacterial properties of both EOs are closely related to their taxonomic characteristics, while their low antioxidant activity is rationalized by considering that they are mainly composed of monoterpene hydrocarbons [10]. In another study, nine compounds were isolated from *C. bulbosum* plants collected from Ardahan (Turkey), and their chemical structures were elucidated using IR, NMR, and MS spectroscopy. The assessment of the antioxidant and enzyme-inhibitory activities of these compounds highlighted *C. bulbosum* as a potent source for the development of plant-based formulations for the food, pharmaceutical and cosmetic industries [11]. *C. coloratum* is *a* less investigated endemic plant of the Western Balkans which is also classified into the *bulbous chervil* plants. Its histochemical analysis and determination of secondary metabolite content revealed that various parts of *C. coloratum* contain EOs differing in both their composition and individual compound contents. Specifically, EO isolated from its roots is characterized by a high content of monoterpene myrcene (72.18%), while monoterpenes *β-(E)-*ocimene (33.59%), *β-(Z)-*ocimene (20.43%) and terpinolene (10.77%) are the prevailing molecules in EO from its stems. On the other hand, the main constituents of EO isolated from its leaves are the molecules spathulenol (10.19%), *p-*cymene-8-ol (9.45%) and *p-*cymene (7.6%), while the monoterpenes terpinolene (17.08%) and *p-(Z)-(E)-*ocimene (6.96%) have been identified as the main constituents of EO isolated from its flowers. Finally, the prevailing constituents of EO isolated from its fruits are the molecules caryophyllene oxide (6.93%), *(Z)-β-*farnesene (5.92%), *(E)-*pinocarveol (5.56%) and myrtenol (5.25%) [12].

According to the Flora of Armenia the following five *Chaerophyllum* species grow in the country: *C. macrospermum (Willd. ex Spreng.) Fisch. & C.A. Mey*, *C. bulbosum* L. (*syn. C. caucasicum (Fisch. ex Hoffm*.) Schischk.), *C. crinitum Boiss., C. roseum Bieb.* and *C. aureum L*. The existence of an additional species, namely *C. humile M. Bieb*, is also reported [13,14].

Although the literature abounds with reports concerning the phytochemical composition and biological activities of *Chaerophyllum* species sampled from different geographical regions, there are no reports on *C. bulbosum* plants growing in Armenia. Therefore, the main objectives of the present study are the determination of the chemical contents of EO and hydrosol isolated from the aerial parts of a *C. bulbosum* plant collected in Armenia and an evaluation of their antimicrobial activity against selected bacterial and fungal microorganisms.

## 2. Results and Discussion

### 2.1. Chemical Contents of C. bulbosum Herb Essential Oil and Hydrosol

Since the gastronomic and nutritional value of flowers has recently attracted considerable research attention, several edible plants (EPs) have been exploited in respect to their attractiveness, sensory perception, nutritional properties, and biological activities. EPs are versatile and suitable for various culinary purposes, and their flowers are used without processing or in powdered form. Furthermore, since their EOs have also attracted increasing interest from the food industry there are numerous scientific reports exploiting their antimicrobial and antioxidant properties. Thus, several EOs are currently used as safer substitutes for chemical preservatives to meet the consumer demand for reductions in artificial and harmful compound utilization by the food industry [15,16]. On the other hand, hydrosols, which are also referred to as aromatic waters, are byproducts obtained during EO production via the hydrodistillation processing of plant material. They consist of distilled water and a small amount of dispersed EO components. The latter are mainly composed of oxygenated compounds, since hydrophobic molecules such as monoterpene and sesquiterpene hydrocarbons are extracted into the EO. Hydrosols constitute the major byproduct of the EO production process and are frequently used by the food, cosmetic and perfume industries [17,18].

Herein, the presence of 48 volatile compounds, accounting for 98.39% of the contained molecules, was determined in the investigated EO of *bulbous chervil* plant species growing wild in Armenia. They consisted of 60.42% monoterpenes and their oxygenated derivatives, 18.75% sesquiterpenes and their oxygenated derivatives, and the remaining 20.83% composed of a diverse class of molecules such as alkanes, aldehydes, diterpene alcohols and other substances. The detailed results are included in Table 1, highlighting the molecules pulegone (21.55%), piperitenone (19.94%), piperitone (9.52%), neomenthol (9.20%) and menthol (5.58%) as the most abundant components of the EO.

Since variables such as climatic conditions, seasonal variation and the plant’s ecotype greatly influence EO composition, the chemical composition of the EO investigated herein greatly differs from that of EOs originating from different geographical regions. Thus, the EO investigated herein contains the molecule pulegone as its prevailing component, while in EO from *C. bulbosum* collected in Serbia, the most abundant molecule is (*E*)-*β*-ocimene (38.5–91.5%). On the other hand, apiol (37%) is the predominant molecule of the corresponding product sampled from Greece, and (*E*)-*β*-farnesene (22.3%) is the major compound of EO from *C. bulbosum* growing in Iran [10].

Pulegone is the most abundant monoterpene molecule in the investigated EO. Its presence in large quantity is of particular importance since this monoterpene ketone has been detected in the EOs of various mint species, such as *Hedeoma pulegoides* and *Mentha pulegium*. These EOs are frequently incorporated into food and beverage preparations as a fragrant and/or flavoring agent [19,20]. The presence of 8,9-dehydrothymol in a lesser amount (1.21%) is also noteworthy, since according to literature data its glycoside derivative 8,9-dehydrothymol-3-O-β-glucoside has been determined to exhibit cytotoxic activity against the MCF-7, HeLa, A549, and Hep G-2 cancer cell lines [21].

In respect of the non-monoterpenoid fraction, the presence of paeonol is notable because of its great therapeutic potential. Paeonol is reported to display multi-spectrum pharmacological activity, including anti-inflammatory, anti-diabetic, neuroprotective, nephroprotective, cardioprotective, and antitumor activities [22,23].

Nevertheless, the monoterpene derivatives that constitute the major fraction of the EO, accounting for 60.42% of its components, are a class of molecules contributing greatly to the prevention and treatment of various diseases. In this context, the development of new structures based on monoterpene compounds is a dynamically developing field, and the results herein may contribute towards this objective.

In respect of the chemical content of the hydrosol, derived as the major byproduct of the EO production procedure, it contains monoterpene oxygenated derivatives (75%), monoterpenes (20%) and a small sesquiterpenoid fraction (5%). The detailed results are included in Table 2, highlighting as its most abundant monoterpenes the molecules pulegone (24.39%), piperitenone (23.80%) and piperitone (11.81%). Among these compounds the presence of the monoterpenoid piperitenone is of particular interest since this molecule is known to produce a wide range of bioactive molecules via diverse chemical pathways [24,25]. On the other hand, piperitone has been detected as a constituent of numerous EOs, including those from over thirty species of the Eucalyptus genus and several Mentha species [26].

In summary, the EO and hydrosol of *bulbous chervil* species growing wild in Armenia are characterized by an entirely different chemical content as compared to the EOs of *bulbous chervil* species grown in other parts of the globe, indicating the existence of a different chemotype.

### 2.2. Total Phenolic Content (TPC), Total Flavonoid Content (TFC) and Antioxidant Activity

Phenolic compounds such as flavonoids and phenolic acids are secondary metabolites of plant organisms increasingly used by the food industry as bioactive food ingredients, since they display the ability to slow down the oxidative degradation of lipids and improve the quality and nutritional value of foods. Flavonoids are naturally present in plants providing a positive effect on human health, since several studies have verified that they exhibit notable antioxidant, antibacterial, anti-inflammatory, anticancer, and anti-allergic activities [27,28,29]. The dried methanolic extract of the *C. bulbosum* plant investigated herein was determined to display modest phenolic and flavonoid contents, with values for TPC of 1.17 ± 0.03 mg/g expressed as gallic acid equivalents (GAE) and for TFC of 0.62 ± 0.02 mg/g expressed as quercetin equivalents (QE).

On the contrary, the evaluation of antioxidant activity determined that the plant extract possessed strong antioxidant potency, displaying IC_50_ = 2.87 ± 0.06 μg Trolox/g dried plant extract for the DPPH^•^ assay and 46.87 ± 1.2 μmol Fe^2+^/g dried plant extract for the FRAP assay.

### 2.3. Antimicrobial Activity of C. bulbosum EO and Hydrosol

Most EOs isolated from medicinal plants exhibit a broad spectrum of well-known antimicrobial and antifungal activities [30,31]. Herein, the antimicrobial properties of *Cyperus bulbosum* EO and hydrosol were evaluated against six bacterial strains and the yeast *Candida albicans* using the disk diffusion method. The respective results are included in Table 3, revealing that both possess inhibitory activity against all tested microorganisms, with their effectiveness varying considerably among the investigated strains.

Among the tested bacterial strains, *Streptococcus mutans* showed the highest susceptibility to both the EO and hydrosol, displaying inhibition zones of 19.5 ± 0.3 mm and 20.0 ± 0.8 mm, respectively. These values are comparable to the inhibition determined for tetracycline (25 ± 0.4 mm), revealing their strong antibacterial effect against this cariogenic bacterium. Similar susceptibility was observed for *Staphylococcus aureus*, with inhibition zones of 15.2 ± 0.8 mm and 14.6 ± 1.3 mm for the EO and hydrosol, respectively.

On the other hand, moderate antibacterial activity was detected against *Enterococcus faecalis* and *Lactobacillus salivarius*, with inhibition zones ranging from 9.8 to 13.8 mm. In contrast, the Gram-negative bacteria *Escherichia coli* and *Pseudomonas aeruginosa* exhibited lower susceptibility, recording inhibition zones below 8.5 mm for both samples tested. Notably, *P. aeruginosa* was the most resistant among all microorganisms tested, with inhibition zones of 7.0 ± 1.3 mm and 6.3 ± 0.6 mm for the EO and hydrosol, respectively.

In respect of the antifungal activity tested against the *C. albicans* strain, the observed activity was moderate, with inhibition zones of 13.0 ± 1.2 mm for the EO and 11.0 ± 0.8 mm for the hydrosol. Although these values are lower as compared to fluconazole’s activity (21 ± 0.8 mm), they are indicative of a clear inhibitory effect against the fungal pathogen.

Overall, the results suggest that both the EO and hydrosol exhibit a broad spectrum of antimicrobial activities, mainly against Gram-positive bacteria, which were determined as being more susceptible as compared to Gram-negative bacteria. This pattern is in line with previous reports [32,33] for plant-derived EOs and is attributed to structural differences in the cell wall: Gram-negative bacteria possess an outer membrane rich in lipopolysaccharides, which form a barrier that limits the penetration of hydrophobic compounds [34].

### 2.4. Minimum Inhibitory Concentration (MIC) and Minimum Bactericidal Concentration (MBC) Values

The antimicrobial potency of the EO and hydrosol was further investigated by determining the MIC and MBC values, which respectively reflect the lowest concentration for no visible microorganism growth as compared to control wells and the lowest concentration resulting in the microorganisms’ destruction, defined by the absence of their growth after subculture from wells with no visible growth. These values were determined in three independent experiments using the broth microdilution method to produce identical endpoint values revealing the results’ consistency across the replicates.

The respective results are included in Table 4, revealing that the EO’s potency was superior as compared to the hydrosol, since the determined MIC and MBC values for the EO were substantially lower as compared to those for the hydrosol against all tested microorganisms. In particular, the observed MIC values for the EO were between 0.4 and 6.0 µL/mL, whereas the respective MBC values ranged from 0.8 to 10.0 µL/mL. The lowest MIC value was observed against *S. mutans* (0.4 mg/mL), followed by *S. aureus* (0.5 µL/mL), confirming the high susceptibility of these Gram-positive bacteria. On the contrary, *P. aeruginosa* displayed the highest MIC (6.0 µL/mL) and MBC (10.0 µL/mL), indicating marked resistance. Finally, the antifungal activity of the EO against *C. albicans* was also notable, displaying MIC and MBC values of 1.00 and 2.00 µL/mL, respectively.

On the contrary, the hydrosol exhibited MIC values ranging from 62.5 to 400 μL/mL and MBC values ranging from 125 to 800 μL/mL. The strongest antibacterial activity was again observed against *S. mutans*, with MIC and MBC values of 62.5 and 125 μL/mL, respectively. The lowest susceptibility was recorded against *E. faecalis*, *L. salivarius*, and *C. albicans*, each requiring MIC and MBC values of 400 and 800 μL/mL, respectively.

## 3. Materials and Methods

### 3.1. Plant Material Collection and Identification

The herb specimen was collected from Aparan region of Armenia (RA) in June 2025 at the beginning of the flowering period. The preparation of the raw material (harvesting, drying, preliminary standardization, etc.) was carried out following the guidelines of good agricultural and collection practices (GACP) for medicinal plants provided by WHO [35]. The plant material processing was meticulously conducted at the Department of Pharmacognosy at YSMU of Armenia (RA), revealing that the specimen was identified as *C. bulbosum* L. and gathered from the geographic coordinates latitude 40°35′35.63″ N and longitude 44°21′32.04″ E, at an elevation of 1889.0 m above sea level (Aparan region of RA). The species identification was performed by the Institute of Botany of the National Academy of Science of Armenia, where a voucher specimen assigned the identifier ERE 192246 has been deposited.

### 3.2. Chemicals and Extractions

The analytical-grade methanol used for the extractions was purchased from Fisher Chemicals (Hampton, NH, USA).

The following reagents and solvents were used for the assessments: 2,2-diphenyl-1-picrylhydrazyl (DPPH), 2,4,6-tris(2-pyridyl)-s-triazine (TPTZ), Folin–Ciocalteu’s reagent and analytical-purity glacial acetic acid purchased from Sigma-Aldrich (Burlington, MA, USA); anhydrous sodium carbonate (99.999+% purity) and sulfuric acid (98% purity), from Chem-Lab; analytical-purity chloroform, diethyl ether, dimethylsulfoxide (DMSO) and petroleum ether (40–60 °C) from Fisher Chemicals (Hampton, NH, USA); heptahydrate iron sulfate (99+% purity), hexahydrate ferric chloride (97% purity), and potassium hydroxide (KOH, 99.99% purity) from Alfa Aesar (Holbrook, NY, USA); trolox (6-hydroxy-2,5,7,8-tetramethylchroman-2-carboxylic acid (95% purity) and vanillin (99% purity) provided by Acros Organics (Geel, Belgium).

### 3.3. Isolation of EO

The EO of *bulbous chervil* was obtained from the aerial parts of the plant by applying the hydrodistillation method Clevenger-type apparatus for a duration of 3 h. The isolated EO was subsequently dried over anhydrous sodium sulfate, while the aqueous fraction (hydrosol) was separately collected. Both samples were stored at 4 °C in light-protected amber/dark glass containers in accordance with established guidelines [36] until further analysis.

### 3.4. Extraction Procedure

All extractions were implemented using an RK 100 SH, Bandelin Sonorex Super (Bandelin, Berlin, Germany) ultrasonic bath. The obtained extracts were evaporated under vacuum and heat-assisted evaporation, at temperatures below 35 °C, using a Büchi Rotavapor R-210 apparatus equipped with a Büchi vacuum pump V-700, vacuum controller V-850 (all obtained from Büchi, Flawil, St Gallen, Switzerland) and Julabo F12 (Seelbach, Germany) cooling unit. Estimation of the TPC and TFC was implemented using an Infinite^®^ 200 PRO microplate reader (Tecan Group Ltd., San Jose, CA, USA).

Specifically, the dried *bulbous chervil* herb was ground and the contained phenolics were extracted according to the following procedure: A 50 g dried, powdered plant sample was added into a 200 mL mixture of MeOH/H_2_O/1.0 N HCl (90:9.5:0.5 *v*/*v*) and sonicated for 10 min in the ultrasonic bath (35 kHz). The solvent was separated by filtration, and the remaining solid was re-extracted two additional times following the same procedure. The extracts were combined, the solvent was removed under reduced pressure, and the remaining solid was dissolved in 50 mL of MeOH/H_2_O (1:1) mixture and centrifuged for 10 min (7500 rpm). The supernatant liquid was extracted with petroleum ether (3 × 30 mL) to remove the contained lipids, and the combined extracts were evaporated under vacuum. The remaining solid was poured into 50 mL of brine and extracted three times with ethyl acetate (EtOAc, 3 × 50 mL) to remove possible sugars dissolved into the aqueous layer. The combined organic layers were dried over anhydrous MgSO_4_ and evaporated under reduced pressure to dryness, yielding a solid, which was analyzed. To avoid polyphenol degradation, all abovementioned activities were performed in the absence of direct sunlight, with temperatures kept below 35 °C.

### 3.5. Gas Chromatography–Mass Spectrometry (GC-MS) Analysis

The chemical composition of the EO and hydrosol obtained from the *C. bulbosum* herb using the hydrodistillation method was determined by GC–MS analysis. For this purpose, the following instrumentation was used: an Agilent Technologies 7890A gas chromatograph equipped with an HP 5MS 30 m × 0.25 mm × 0.25 μm film thickness capillary column and connected with an Agilent 5957C, VL MS Detector with a Triple-Axis Detector (all obtained from Agilent Scientific Instruments, Santa Clara, CA, USA). The MS system was operated in Electron Impact mode with the electron energy set at 70 eV and a scan range of 29–380 *m*/*z*. Helium was used as the carrier gas at a constant flow rate of 1 mL/min, while sample injection was performed using an autosampler. The column temperature was initially set at 60 °C and increased gradually to 280 °C at a 3 °C/min rate.

The identification of the contained compounds was performed by comparing their: (i) linear retention indices (LRIs) calculated by using a homologous series of even-numbered n-alkanes (C8–C24) with those of standard compounds and the respective literature data [37], and (ii) EI-MS spectra with those of reference compounds and the respective spectral MS data obtained from the NIST 11 and Willey 275 libraries [38,39].

### 3.6. Determination of Total Phenolic (TPC) and Total Flavonoid (TFC) Contents

The TPC and TFC assessments were implemented using an Infinite^®^ 200 PRO microplate reader (Tecan Group Ltd., San Jose, CA, USA), according to the following procedures: The TPC was determined as follows: A 10 µL portion of methanolic solution of each sample diluted in 100 µL of water and 10 µL of the Folin–Ciocalteu reagent solution was added in triplicate to a 96-well microplate (Sarstedt AG & Co. KG, Nümbrecht, Germany). After incubation for 3 min at room temperature, 20 µL of an aqueous solution of sodium carbonate (7.5% *w*/*v*) and 60 µL of water were successively added, and the incubation was continued in the dark for an additional period of 60 min. Each sample’s absorption was measured at 765 nm wavelength, and the measurement was utilized for TPC determination against a standard calibration curve previously prepared with 30–180 µg/mL solutions of gallic acid in methanol. The results were obtained by applying the equation y = 0.0017x + 0.0296 (R^2^ = 0.9982), where y represents the absorbance at 765 nm and x is the concentration of gallic acid in μg/mL. The TPC values are expressed as mg of gallic acid equivalents (mg GAE) per g of dried plant material [40].

The TFC value was measured using a modified version of the aluminum chloride method developed by Pekal and Pyrzynska [41]. Specifically, 100 µL of each sample was mixed with 50 µL of aluminum chloride (AlCl_3_) aqueous solution (2% *w*/*v*) and 50 µL of sodium acetate (CH_3_COONa) aqueous solution (1 M), and the mixture was placed in a 96-well microplate. After incubation at room temperature in the absence of light for 40 min, the absorbance was measured at 415 nm using a microplate reader. The TFC value was calculated against a standard calibration curve of quercetin in methanol of various concentrations and y = 0.0118x−0.0577 (R^2^ = 0.9981), where y represents the absorbance at 415 nm and x is the concentration of quercetin in μg/mL. The TFC values are expressed as mg of quercetin equivalents (mg QE) per g of dried plant material [41].

### 3.7. Antioxidant Property Evaluation

The FRAP (Ferric Reducing Antioxidant Power) and DPPH^•^ (Radical Scavenging Assay) procedures were utilized for the estimation of the investigated samples’ antioxidant capacities. Both assessments were conducted using a NanoQuant infinite M200PRO (Tecan Group Ltd., Männedorf, Switzerland) instrument following previously reported procedures [14]. Specifically, the absorbance for the FRAP assay was measured at 593 nm wavelength, and the reducing capacity was determined against an FeSO_4_ standard calibration curve. The obtained results are expressed as mmol Fe^2+^/g of extract. Accordingly, the absorbance for the DPPH^•^ assay was measured at 515 nm wavelength, and the antioxidant activity was determined against a Trolox calibration standard curve. The results are expressed as mg Trolox Equivalents (TE)/g of extract [42].

### 3.8. Determination of Antimicrobial Activity, MIC, and MBC of EO and Hydrosol of C. bulbosum

The antimicrobial activity of *C. bulbosum* essential oil and hydrosol was evaluated using agar disk diffusion and broth microdilution methods according to CLSI and EUCAST guidelines. Six test bacteria and *C. albicans* were selected to assess antimicrobial activity: *Escherichia coli* ATCC 8739, *Enterococcus faecalis* ATCC 29212, *Staphylococcus aureus* ATCC 25923, *Streptococcus mutans* ATCC 35668, *Lactobacillus salivarius* ATCC 11741, *Pseudomonas aeruginosa* ATCC 27853 and *Candida albicans* MDC 10002. All abovementioned microbial cultures were obtained from the Microbial Depository Center (MDC) of SPC “Armbiotechnology” of the National Academy of Sciences of Republic of Armenia, and experiments were carried out at the Laboratory of Alternative Energy Sources at the Center. The cultures were stored at 4 °C on agar slides and subcultured monthly. For cultivation of the microorganisms, Nutrient broth (NB) (Condalab, Torrejón de Ardoz, Spain), Mueller–Hinton agar and broth (Liofilchem, Italy) and Sabouraud Dextrose Agar (SDA) (HiMedia, India) were used.

For the agar disc diffusion method, overnight microbial cultures were diluted with a 0.9% NaCl solution to achieve a concentration of 0.5 McFarland, resulting in 108 CFU/mL. Subsequently, 0.1 mL of this dilution was spread over the Mueller–Hinton agar surface. The culture plates were incubated for 24 h at 37 °C for bacteria and 48 h at 28 °C for *Candida albicans*.

Inhibition zones were determined by measuring the diameter (mm) of the clear growth inhibition area surrounding sterile paper discs (6 mm diameter; Condalab, Torrejón de Ardoz, Spain) impregnated with 10 µL of essential oil or 20 µL of hydrosol per disc. The control antibiotics were tetracycline 30 µg and fluconazole 25 µg (Condalab, Torrejón de Ardoz, Spain). Discs impregnated with 10% DMSO were used as a negative control. They did not produce inhibition zones against any of the tested bacterial or fungal strains, indicating that the observed antimicrobial activity in the MIC and MBC tests was solely attributable to the essential oil and hydrosol, not the solvent DMSO.

The MIC values of the essential oil and hydrosol were determined using the broth microdilution method according to CLSI guidelines with minor modifications. The essential oil was dissolved in 10% DMSO and serially diluted two-fold in Mueller–Hinton broth to obtain final concentrations ranging from 0.1 to 100 μL/mL. Hydrosol samples were serially diluted with sterile broth to obtain concentrations ranging from 15.6 to 1000 μL/mL.

Fresh microbial suspensions were adjusted to 0.5 McFarland standard and further diluted to obtain a final inoculum of approximately 5 × 10^5^ CFU/mL in each well. Aliquots of 100 μL of diluted sample and 100 μL of microbial suspension were added to sterile 96-well microplates.

Positive growth controls contained inoculated broth without test samples, while negative controls contained sterile broth only. Solvent controls containing the highest concentration of DMSO used in the assay were included to verify the absence of antimicrobial effects of the solvent.

The microplates were incubated at 37 °C for 24 h for bacterial strains and at 28 °C for 48 h for *Candida albicans*. MIC values were defined as the lowest concentration showing no visible microbial growth compared with the control wells.

### 3.9. Statistical Analysis

Statistical comparisons between groups within each experiment were implemented using one-way analysis of variance (ANOVA). All experiments were performed in triplicate with two parallel measurements (*n* = 3).

## 4. Conclusions

This study represents the first comprehensive phytochemical and antimicrobial investigation of *C. bulbosum* L. growing in Armenia. The GC–MS analysis revealed a distinct chemotype dominated by the presence of pulegone, piperitenone, and piperitone phytochemicals, markedly differentiating this specimen from previously reported populations from other geographical regions. These findings highlight the influence of environmental and ecological factors on the secondary metabolite profile of the species.

Both the EO and hydrosol demonstrated pronounced antimicrobial activity, particularly against Gram-positive bacteria, with *Streptococcus mutans* and *Staphylococcus aureus* being the most susceptible microorganisms. Additionally, the EO exhibited stronger activity as compared to the hydrosol, evidenced by lower MIC and MBC values, while both preparations showed moderate antifungal activity against *Candida albicans*. The observed antimicrobial activity is presumably associated with the high abundance of oxygenated monoterpenoids, especially pulegone, piperitenone, piperitone, menthol, and related compounds. In addition, the presence of phenolic and flavonoid constituents renders *C. bulbosum* EO a potent antioxidant, highlighting the herb as a rich source of bioactive phytochemicals.

Overall, the results herein reveal that the Armenian populations of *C. bulbosum* represent a promising natural source of antimicrobial agents and merit further investigation regarding their antioxidant properties, safety profile, mechanisms of action, and potential applications in pharmaceutical, cosmetic, and food-preservation products.

## Figures and Tables

**Table 1:** Volatile constituents detected in the EO of *C bulbosum* L.

No.	Compound	Formula	Name and Identifier	Rt ^a^	% ^b^
1	*β*-Myrcene	C_10_H_16_	monoterpene	14.43	1.04
2	*o*-Cymene	C_10_H_14_	aromatic hydrocarbon	16.01	0.19
3	Eucalyptol	C_10_H_18_O	monoterpenoid	16.24	0.27
4	*trans*-*β*-Ocimene	C_10_H_16_	monoterpene	16.84	1.98
5	*β*-Ocimene	C_10_H_16_	monoterpene	17.35	1.49
6	*γ*-Terpinene	C_10_H_16_	monoterpene	17.77	0.24
7	*p*-Mentha-3,8-diene	C_10_H_16_	monoterpene	18.35	0.19
8	Paeonol	C_9_H_10_O_3_	phenol	19.63	0.16
9	Linalool	C_10_H_18_O	monoterpenoid	19.95	0.37
10	2-Heptanone, 6-methyl	C_8_H_16_O	-	20.15	0.34
11	*Allo*-Ocimene	C_10_H_16_	monoterpene	21.37	1.82
12	*Cis*-Myroxide	C_10_H_16_	monoterpene	21.57	0.58
13	Myroxide	C_10_H_16_	monoterpene	22.03	0.30
14	1-Cyclohexene-1-methanol, α,α,4-trimethyl-	C_8_H_16_O	monoterpenoid	22.19	3.08
15	*trans*-(–)-*p*-Menthan-3-one	C_8_H_16_O	monoterpenoid	22.40	3.07
16	*p*-Menthan-3-one	C_8_H_16_O	monoterpenoid	22.90	3.35
17	Neomenthol	C_10_H_20_O	monoterpenoid	22.97	9.20
18	*p*-Mentha-1,5-dien-8-ol	C_10_H_16_O	monoterpenoid	23.09	1.52
19	Levomenthol	C_10_H_20_O	monoterpenoid	23.38	0.43
20	Terpinen-4-ol	C_10_H_18_O	monoterpenoid	23.52	1.66
21	Menthol	C_10_H_20_O	monoterpenoid	24.14	5.58
22	1-Cyclohexylethanol	C_8_H_16_O	monoterpenoid	24.77	0.13
23	Cosmen-2-ol	C_8_H_16_O	monoterpenoid	25.23	0.99
24	8,9-Dehydrothymol	C_10_H_12_O	monoterpenoid	25.49	1.21
25	Pulegone	C_10_H_16_O	monoterpenoid	26.49	21.55
26	Piperitone	C_10_H_16_O	monoterpenoid	27.16	9.52
27	Geraniol	C_10_H_18_O	monoterpenoid	27.41	0.74
28	*p*-Mentha-1,8-dien-3-one, (+)-	C_10_H_14_O	monoterpenoid	28.00	0.65
29	3-Hexen-1-ol, 2-ethyl	C_8_H_16_O	-	28.21	0.50
30	6-Ethyl-2-methyloctane	C_11_H_24_	aliphatic hydrocarbon	28.52	0.13
31	Car-3-en-5-one	C_10_H_14_O	monoterpenoid	29.04	0.14
32	Thymol	C_10_H_14_O	monoterpenoid	29.17	1.14
33	Piperitenone	C_10_H_14_O	monoterpenoid	31.06	19.94
34	2-Decyn-1-ol	C_10_H_18_O	monoterpenoid	31.51	0.34
35	Geranyl acetate	C_12_H_20_O_2_	monoterpenoid	33.10	0.77
36	Humulene	C_15_H_24_	sesquiterpene	35.78	0.18
37	(*E*)-*β*-Farnesene	C_15_H_24_	sesquiterpene	36.10	0.10
38	Germacrene-D	C_15_H_24_	sesquiterpene	36.92	0.44
39	*trans*-*β*-Ionone	C_13_H_20_O	terpene ketone	37.19	0.27
40	Hexadecane	C_16_H_34_	hydrocarbon	37.66	0.17
41	Myristicin	C_11_H_12_O_3_	phenylpropene	38.70	0.24
42	Spathulenol	C_15_H_24_O	sesquiterpenoid	40.69	1.05
43	Caryophyllene oxide	C_15_H_24_O	sesquiterpenoid	40.88	0.49
44	Humulene oxide	C_15_H_24_O	sesquiterpenoid	41.89	0.33
45	(+)-*α*-Bisabolol	C_15_H_26_O	sesquiterpenoid	43.63	0.26
46	Heptadecane	C_17_H_36_	alkane hydrocarbon	45.72	0.14
47	Bisabolol oxide	C_15_H_26_O_2_	sesquiterpenoid	46.87	0.33
48	Phytol	C_20_H_40_O	diterpene	58.84	0.12
	TOTAL				98.39

^a^ Rt Retention time. ^b^ %: Percentage of the total peak area. Only components with percentage *n* ≥ 0.10% are presented.

**Table 2:** Volatile constituents detected in the *Chaerophyllum bulbosum* L. hydrosol.

No.	Compound	Formula		Rt ^a^	% ^b^
1.	*β*-Myrcene	C_10_H_16_	monoterpene	14.43	1.11
2.	*trans-β*-Ocimene	C_10_H_16_	monoterpene	16.84	1.75
3.	β-Ocimene	C_10_H_16_	monoterpene	17.35	1.35
4.	Allo-Ocimene	C_10_H_16_	monoterpene	21.38	1.97
5.	1-Cyclohexene-1-methanol, α,α,4-trimethyl	C_10_H_18_O	monoterpenoid	22.19	2.78
6.	*Trans*-(–)-*p*-Menthan-3-one	C_10_H_18_O	monoterpenoid	22.40	2.76
7.	*p*-Menthan-3-one	C_10_H_18_O	monoterpenoid	22.90	3.35
8.	Neomenthol	C_10_H_20_O	monoterpenoid	22.97	9.27
9.	*p*-Mentha-1,5-dien-8-ol	C_10_H_16_O	monoterpenoid	23.09	1.52
10.	Levomenthol	C_10_H_20_O	monoterpenoid	23.38	0.62
11.	Terpinen-4-ol	C_10_H_18_O	monoterpenoid	23.52	1.55
12.	Menthol	C_10_H_20_O	monoterpenoid	24.15	5.60
13.	Cosmen-2-ol	C_10_H_18_O	monoterpenoid	25.23	0.74
14.	8,9-Dehydrothymol	C_10_H_12_O	monoterpenoid	25.49	1.33
15.	Pulegone	C_10_H_16_O	monoterpenoid	26.49	24.39
16.	Piperitone	C_10_H_16_O	monoterpenoid	27.16	11.81
17.	Geraniol	C_10_H_18_O	monoterpenoid	27.41	0.71
18.	Thymol	C_10_H_14_O	monoterpenoid	29.16	1.19
19.	Piperitenone	C_10_H_14_O	monoterpenoid	31.06	23.80
20.	Spathulenol	C_15_H_24_O	sesquiterpenoid	40.69	1.05

^a^ Rt: Retention time. ^b^ %: Percentage of the total peak area. Only components with percentage *n* ≥ 0.10% are presented.

**Table 3:** Growth inhibition zones of *C. bulbosum* EO and hydrosol against tested strains, determined by disk diffusion method.

Test Strains	Hydrosol Zone (mm)	Essential Oil Zone (mm)	Fluconazole 25 µg, (mm)	Tetracycline 30 µg, (mm)
*Str. mutans*	20.0 ± 0.8	19.5 ± 0.3	-	25 ± 0.4
*St. aureus*	14.6 ± 1.3	15.2 ± 0.8	-	26 ± 1.2
*Ent. faecalis*	13.8 ± 1.5	13.5 ± 1.1	-	16 ± 0.8
*E. coli*	8.1 ± 0.4	7.2 ± 1.0	-	22 ± 0.7
*P. aeruginosa*	6.3 ± 0.6	7.0 ± 1.3	-	8 ± 1.0
*L. salivarius*	9.8 ± 1.2	10.6 ± 0.5	-	9.5 ± 1.1
*C. albicans*	11 ± 0.8	13 ± 1.2	21 ± 0.8	-

“-” represents no antimicrobial activity observed at tested concentrations. Values are expressed as mean ± SD of three independent experiments (*n* = 3). Statistically significant differences were observed in data.  p<0.05.

**Table 4:** The minimum inhibitory concentrations (MICs), minimum bactericidal concentrations (MBCs), and minimum fungicidal concentration (MFC) of essential oil (EO) and hydrosol from *C. bulbosum* against tested microorganisms.

Test Strains	Hydrosol (µL/mL)		Essential Oil (µL/mL)	
	MIC	MBC	MIC	MBC
*Str. mutans*	62.5	125	0.4	0.8
*St. aureus*	200	400	0.5	1.00
*Ent. faecalis*	400	800	1.00	2.00
*E. coli*	160	320	2.00	4.00
*P. aeruginosa*	320	640	6.00	10.00
*L. salivarius*	400	800	1.5	3.00
*C. albicans*	400	800	1.00	2.00

All experiments were performed in triplicate with two parallel measurements (*n* = 3).

## Data Availability

The original contributions presented in this study are included in the article. Further inquiries can be directed to the corresponding author.
